# Supplementary figures and images for: Investigation of the Relationship between the S1 Domain and Its Molecular Functions Derived from Studies of the Tertiary Structure
Source: Molecules. 2019 Oct 13;24(20):3681. doi: 10.3390/molecules24203681 (PMC6832287; doi:10.3390/molecules24203681)

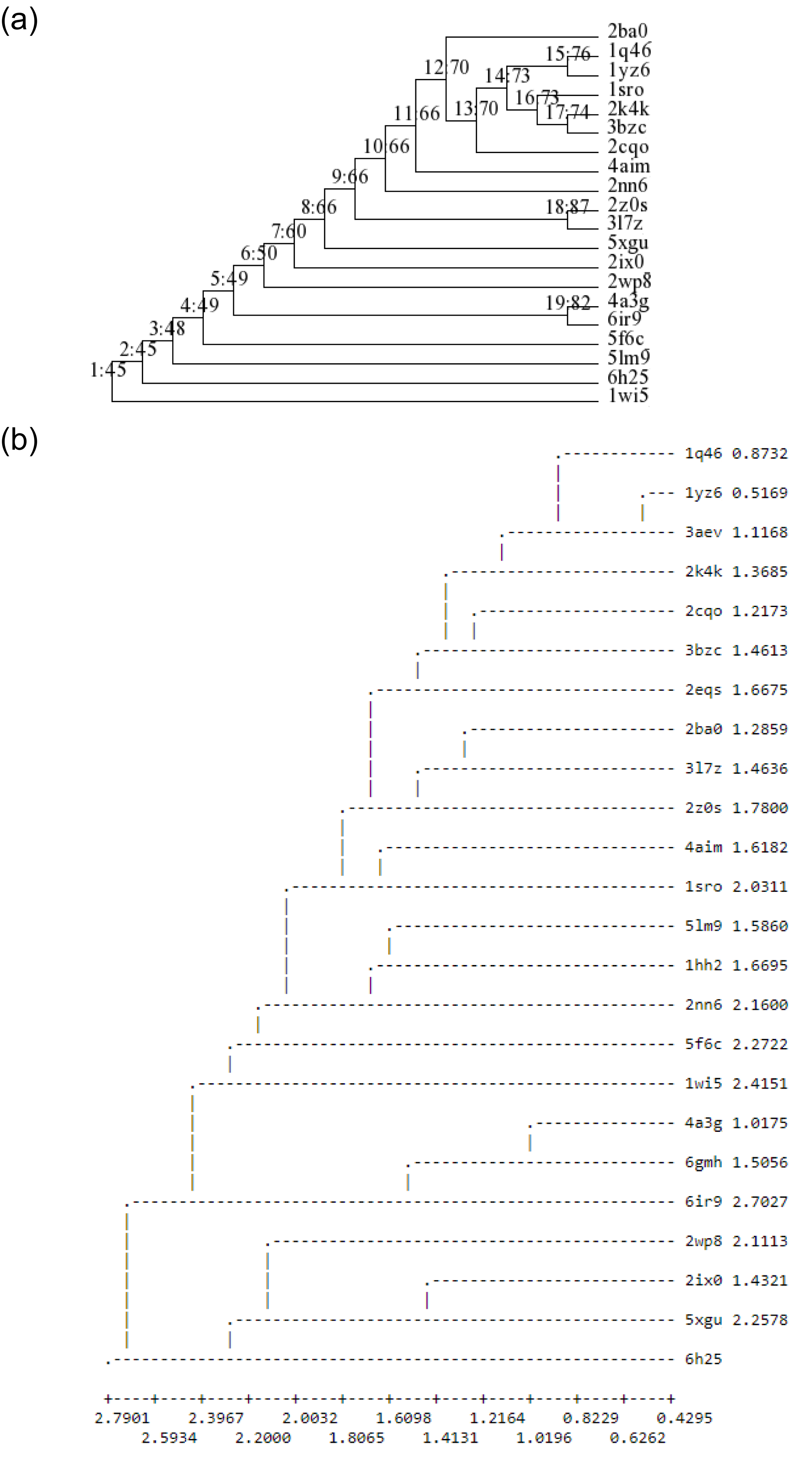

Supplement: Supplementary file 1 [file molecules-24-03681-s001.zip › fig s1.tif]

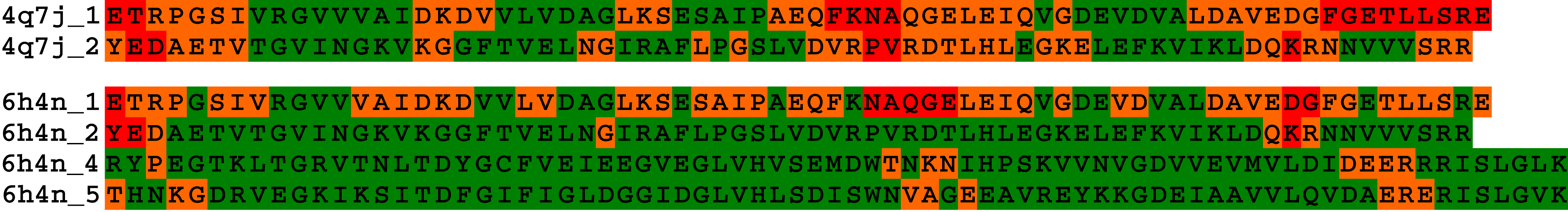

Supplement: Supplementary file 1 [file molecules-24-03681-s001.zip › fig s2.tif]
